# Optimization of alternative breeding schemes for the genetic improvement of common Tigray highland sheep in northern Ethiopia

**DOI:** 10.1186/s12711-022-00755-1

**Published:** 2022-09-16

**Authors:** Kiflay Welday Haileselassie, Solomon Abgaz Kebede, Mengistu Ugre Letta, Solomon Gizaw GebreMichael

**Affiliations:** 1 Department of Animal and Range Sciences, Jig-Jiga University, P.O. Box 1020, Jigjiga, Ethiopia; 2 Ethiopian Institute of Agricultural Research (EIAR), P.O. Box 2003, Addis Ababa, Ethiopia; 3 Schools of Animal and Range Sciences, Haramaya University, P.O. Box 138, Dire Dawa, Ethiopia; 4 International Livestock Research Institute (ILRI), P.O. Box 5689, Addis Ababa, Ethiopia

## Abstract

**Background:**

Genetic improvement is one of the major means to enhance the productivity of livestock, and well-designed animal genetic improvement schemes are necessary to achieve genetic gains. The objective of the current study was to design an alternative breeding program to improve the productivity of common Tigray highland sheep. Two village- and two central nucleus-based breeding schemes were simulated and evaluated in terms of genetic gain, bio-economic efficiency and operational feasibility.

**Methods:**

Four breeding schemes were simulated: scheme 1, a one-tier cooperative village-based breeding scheme, scheme 2, a two-tier cooperative village breeding scheme (dispersed village-based nuclei scheme), scheme 3, a central open nucleus-based scheme with 5% nucleus size; and scheme 4, a three-tier breeding schemes (central open nucleus-based linked with multiplier units). Simulation analyses were performed using the ZPLAN computer program, which is based on a deterministic approach to estimate genetic and economic gains in the breeding programs.

**Results:**

Between the two village-based breeding schemes, scheme 2 showed higher genetic gain and economic efficiency in the breeding traits analysed than scheme 1. The central nuclei schemes were more efficient than the village breeding schemes. Scheme 3 was the most efficient with a genetic gain in the breeding objective of US $ 1.03 and a profit of US $ 2.73/ewe/year, but operationally it is also the most difficult to implement as it requires a big central nucleus. A central nucleus linked with the village-based nuclei would be a feasible option to overcome the operational difficulties of the conventional central nucleus schemes. If a village-based breeding program is considered it should be the first step in most low-input systems. In this case, scheme 2 is the most efficient.

**Conclusions:**

With some support from the public sector at the outset and a strong collaboration among the stakeholders including smallholders, scheme 4 could lead to substantial genetic gains in the common Tigray highland sheep breed within its habitat that covers large areas of the Tigray region. Therefore, we recommend a long-term breeding program which should include cross-breeding, genomic selection, updated estimates of genetic and economic values for the common Tigray highland sheep breed.

## Background

Although common Tigray highland sheep play multiple important roles in the smallholders’ sheep farming systems in the Tigray region of Ethiopia, there have been very few successful and sustained genetic improvement programs. Genetic improvement of livestock is a particularly powerful tool to enhance their productivity. It involves the identification of the breeding objectives, the derivation of economic values for the traits, the design of appropriate schemes, which define the population structure, gene flow, selection strategies, and the development and implementation of the breeding plan [1]. Livestock production in developing regions is generally characterized by small flock sizes, communally shared grazing, uncontrolled mating, multiple and diverse breeding goals, and also the absence of pedigree and performance recording, which limits the development of effective genetic improvement programs.

The current designs of breeding strategies that are suitable for smallholder farming systems can be categorized into village-based and central nucleus-based breeding schemes. The absence of farmers or their minimal participation in the design and implementation of the breeding schemes and inadequate infrastructure have been found to render breeding programs unsuccessful in a low-input production system [2]. To overcome these challenges, village-based breeding schemes have been designed and applied for these systems [3–6].

Village-based breeding programs are intended to overcome the problems of genotype–environment interactions by avoiding the genetic lag between the animals in the nucleus and village populations and to be suitable for the in-situ conservation of indigenous animal genetic resources. Village-based programs also help to bridge the gaps between the skills of breeders (nucleus centers or breeding companies) and farmers to ensure property rights of farmers for improved genetic material. However, the effectiveness of such a design depends on the commitment of the villagers to participate in the program. Failure or lack of compliance by some villagers will affect the level of controlled breeding and the genetic gain that could be achieved [1]. The effectiveness of a village-based breeding program in terms of realized genetic progress and rate of inbreeding is determined by the village flock size that is likely to vary across the breeding tract of a breed that is targeted for improvement. Typically, the common Tigray highland sheep, has a medium to short thin tail and medium to small body size, is widely distributed throughout the region, and contributes in multiple ways to the sustainment of rural livelihoods by producing a wide range of products (meat, manure, wool and stock replacement). It is important to explore alternative breeding strategies to improve the overall sheep productivity and their utilization under the smallholder production system in this region. To optimize breeding schemes, it is necessary to take both the short-term (high rate of genetic gain) and long-term (maintenance of genetic variability and avoidance of inbreeding depression) effects of selection decisions into account [7, 8]. Optimization of the smallholder sheep breeding programs at the community level requires the evaluation of alternative schemes to predict the genetic gain, economic return and operational feasibility during its implementation. This provides an opportunity to adjust the technical, infrastructural and socio-economic issues ahead of the implementation. A ZPLAN computer program has been designed to optimize selection strategies in livestock breeding programs by calculating annual genetic gain for the breeding objectives and return on the investment period [9]. The aim of our study was to evaluate alternative breeding schemes for common Tigray highland sheep in terms of biological aspects, economic efficiencies and the operational feasibility of their implementation under smallholder sheep farming systems. The simulations were based on comprehensive studies of the breeding objectives and production systems of common Tigray highland sheep that were derived from a survey [10] and from monitoring studies.

## Methods

### Description of the study areas

Modelling of the breeding program targeted seven districts of the Tigray Regional State, in northern Ethiopia, which represent different agro-ecological conditions in which the common Tigray highland sheep are bred. The study areas lie within 12° 20′ and 14° 16′ N latitude and 39° 15′ and 39° 45′ longitude. The mean annual temperature ranges from 14 to 22 °C, while the mean annual rainfall ranges from about 400 to 970 mm [11]. Most of the study areas are characterized by a bi-modal rainfall pattern with the main rainy season between June and mid-September and an erratic and unreliable short rainy season in March and April. Common Tigray highland sheep are reared for income generation, breeding (stock replacement) and household consumption.

### Description of production systems

The dominant farming system across all study areas is a mixed crop-livestock production system. Like other parts of Ethiopia, the livestock production system in the study areas is subsistence-oriented, which is characterized by minimal inputs. A low-input production system is found in all livestock production systems prevailing in the country except in the peri-urban and urban systems [12]. Traditional breeding practices vary among farmers, depending on their breeding skills and socio-economic needs. The primary reason for keeping sheep is to generate income, but they are also used for stock replacement, home meat consumption, social security, holiday ceremony and production of manure. The common breeding practice is random breeding, i.e. without clear and consistent selection criteria and with several breeding rams being used in a flock. Faster growing young males are sold because they fetch higher market prices to fulfill immediate family and farmer cash needs such as medical and school fees. Across the study areas, we found no report of a controlled mating system with rams and ewes left together throughout the year. In both the Atsbi-Wemberta and Mekelle districts, mixing adjacent sheep flocks immediately after crop harvests (communal grazing) is the common agricultural practice.

### Breeding objectives and selection criteria

In the current simulation study, we considered the common Tigray highland sheep farmers’ breeding traits as defined previously in [10]: 6-month weight (SMW), twinning rate (litter size), pre-weaning lamb survival (PWLS), 3 to 6 month average daily body weight gain (ADG), lambing interval, and mature body weight. Qualitative traits (coat color, libido, tail type and horn condition) were excluded from the simulation because of their subjective nature. Pre-weaning lamb survival measures the mothering ability of the ewe (i.e., the ability of the dam to raise lambs) while litter size measures the number of lambs born per ewe per lambing. The bio-economic model relating the different breeding objectives included performance traits (6-month weight, average daily body weight gain (3 to 6 months), mature weight, litter size, pre-weaning lamb survival (0 to 3 months) and lambing interval) and economic components (feed, health care, management and fixed costs) and was constructed using a tool that is implemented in a Microsoft Excel sheet for estimating economic values of traits used to design small ruminant breeding programs in the case of smallholder farming practices [13] and that takes both tangible and intangible benefits into account. All the inputs required for estimating bioeconomic weights were derived from interviews with farmers, observations (from September to December 2018), and on-farm performance evaluation of common Tigray high land sheep [10]. The intangible benefits were estimated following Bosman et al. [14]. The economic value of each trait in terms of profit was estimated by the value of an increase of one genetic standard deviation of the trait due to selection. The estimated economic values for litter size, pre-weaning lamb survival, average daily body weight gain (3 to 6 month) and 6-month weight are in Table 2.

### Description of alternative breeding scenarios

Two alternative breeding designs that are increasingly advocated for traditional production systems [15] were considered: a central open nucleus scheme, which is planned and implemented by the regional research institute (Tigray Agricultural Research Institute) and a village-based cooperative breeding program which is implemented by the smallholder village cooperatives.

Each of these two designs consist of two scenarios (schemes): schemes 1 and 2 as village-based breeding schemes and schemes 3 and 4 as conventional (nucleus) breeding programs. These schemes address genetic improvement activities either at the village level (schemes 1 and 2) or at the regional level that covers all of the common Tigray highland sheep breeding tracts (schemes 3 and 4). Seven villages/*kebeles* (four from Atsbie-Wemberta and three from Mekelle area) representing smallholder sheep production systems were selected based on the distribution of this sheep breed, agro-ecological condition, sheep population, accessibility and willingness of the community to participate in the village-based breeding program. Households with at least five breeding ewes were included as members of the breeding cooperative program. Villages are defined as smallholder farmers’ cooperative groups with approximately 2100 breeding ewes (300 ewes per village/*kebele*) as one breeding unit in the breeding program.

The population of common Tigray highland sheep in the seven districts is estimated to be 419,074 individuals. Based on the previous studies [10], the estimated number of animals to be considered as a base population for establishing an open central nucleus is 220,098 and includes 52.52% reproductive ewes. Information on reproductive and production performances was mainly obtained from both field surveys and on-farm monitoring studies of common Tigray highland sheep. Breeding traits were evaluated using a choice card experiment and a bio-economic model. The four schemes were simulated using the ZPLAN computer program [9], which uses a deterministic approach for gene flow and selection index procedures to simulate breeding plans. Based on genetic, biological and economic variables, ZPLAN calculates the genetic gain for the aggregate breeding value, the annual response for each trait and the profit per ewe due to selection. Profit is calculated as the difference between costs and returns.

#### Scheme 1

Scheme 1 is designed to address genetic improvement within a village (i.e., within the flock structure), and is a one-tier cooperative village-based breeding scheme that involves cooperation among smallholder farmers. Selection is carried out in the whole village sheep population. Breeding rams are selected by the farmers from all the flocks in the village that are grouped as one breeding flock and use the selected rams communally. Three hundred ewes per village from smallholder farmers’ cooperative groups and seven *kebeles* were considered as one breeding unit in the breeding program. All the male lambs in the cooperation flocks are recorded and all 6-month old ram lambs in the villages are evaluated as candidates. Selection of the breeding stock in the village flocks is based on the selected candidates’ own 6-month weight and their dams’ lamb rearing record.

#### *Scheme 2*

Since scheme 1 will not show how the genetic improvement within one village can be extended to the different areas where common Tigray highland sheep are raised, scheme 2 is designed to scale up the genetic improvement from two villages/*kebeles* nuclei to the whole common Tigray highland sheep population in the region. It is a two-tier cooperative village breeding scheme (dispersed village-based nuclei scheme) that involves more nuclei breeding villages/*kebeles* where genetic improvement is generated. These serve as sources of improved rams to the whole population of common Tigray highland sheep. The village nuclei are located strategically across the common Tigray highland sheep breeding tract.

#### *Scheme 3*

Scheme 3 is a conventional central open nucleus-based breeding scheme (5%) which is developed by the Regional Agricultural Research Institute (Tigray Agricultural Research Institute) and in which genetic improvement is generated in a central nucleus flock that supplies improved rams to the villages. The nucleus size is a major factor for determining the level of genetic progress and profitability of a scheme. Open nucleus breeding systems require that farmers of the base population do some selection on their female progeny. A very important feature of open nucleus breeding systems is that favorable adaptation genes and other breeding preferences of ‘base’ farmers are secured in the males that are obtained from the nucleus since they are born from what the ‘base’ farmers considered as the ‘best’ females. This feature is a tangible participation of the ‘base’ farmer in the definition of the breeding objectives. The population of nucleus breeding ewes comes from 5% of the total population of ewes. Rams that are used as sires in the base population are selected from a subset of rams that did not qualify for inclusion in the nucleus flock based on best linear unbiased prediction (BLUP) estimated breeding values. Selection in the nucleus is based on selection criteria that are applied in the central nucleus with the involvement of data recorders and animal breeding and genetics experts.

#### *Scheme 4*

**Fig.1:**
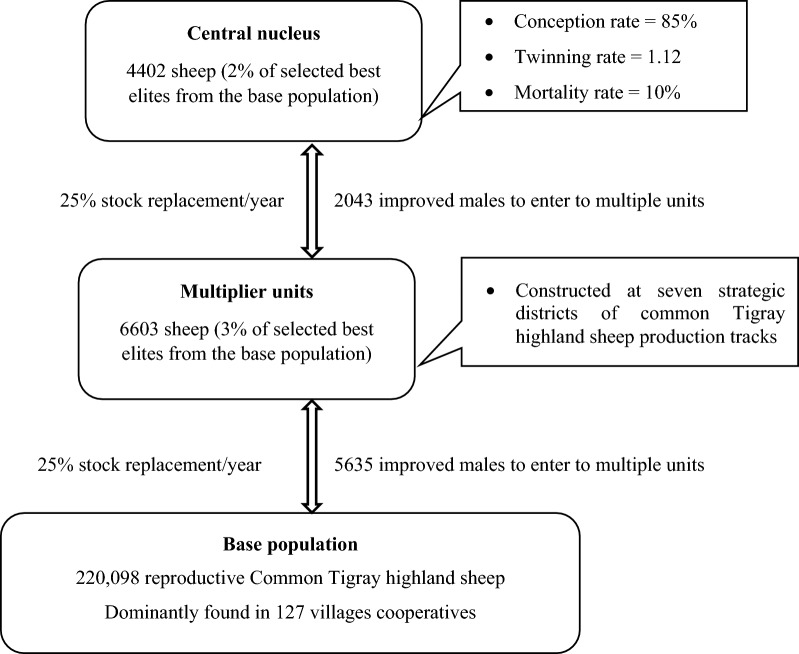
Diagram of three tier breeding schemes for common Tigray highland sheep

Scheme 4 is a three-tier breeding schemes (open breeding nucleus linked with multiplier units). Multi-tiered breeding structures are characterized by the presence of multiple livestock population, which generate, transfer and receive continuous and cumulative genetic improvement [16]. A three-tier breeding scheme is modeled with a 5% nucleus size (2% in the central nucleus and 3% in the multiplier units which are proportionally distributed to the seven village nuclei to address the whole population of common Tigray highland sheep). The central nucleus is linked to village-based multiplier nuclei. The village nuclei (Fig. 1) are operated by village cooperatives breeding groups. A three-tiered gene-flow model was developed to conduct the simulations. The model was based partly on the methodology proposed by Amer [17] who used discounted gene-flow principles to quantify the economic value of the genetic superiority in individual rams or ewes making a one-way genetic contribution to the smallholder production tier. Because of the extent of the genetic contribution and the structure of the breeding program, it is necessary to consider the time and frequency of the expression of genetic superiority in the resulting daughters, the reproductive rates and the culling and survival rates [18].

Multiplier units serve as a bridge to transfer and multiply the superior rams to the population that is located near the village cooperatives, and this requires finding a compromise for both genetic superiority and overall management practice at the farm level. After their establishment, the multiplier units supply superior rams from the central nucleus and from the base population. The elite rams required for the nucleus and multiplier tiers are sourced from the nucleus. Ram lambs born and selected in the multiplier tier are used as sires of lambs in the smallholder farmers. Young female candidates are selected based on their phenotypic performance from the recorded multiplier tiers which produced their own replacement ewes. Replacement nucleus ewes are sourced from within the nucleus and multiplier tiers. The smallholder farmers produced their own ewe replacements that are based on traditional non-recording methods. The gene flow and its expressions from the multiplier rams to the base population are easily adapted to the smallholders’ production system. [19]. Selection of female breeding stock is practiced in the central nucleus and village-based nuclei. The genetic response to selection was calculated as the change in breeding value of the progeny generation over their parent generation.

### Input parameters

Input parameters for the simulation studies are in Table 1. The number of villages and the proportions of the population of the production unit (0.95–1.00) and nucleus (0.0095–0.05) varied from one breeding scheme to another (Table 1). The mating ratio in the village-based breeding schemes (schemes 1 and 2) and nuclei breeding schemes (schemes 3 and 4) were 1:35 and 1:25, respectively. Seven village nuclei (one nucleus per district) were established based on the interests of the smallholder farmers in each district. Population parameters were calculated based on the initial number of ewes, in each respective village. The number of male candidates was calculated as the initial number of ewes, conception rate, twinning rate, number of parturitions per year, survival rate for 3-months, lambing rate and sex ratio. The number of breeding ewes was assumed to be the equal to the sum of the initial number of ewes and the number of candidate females since the initial ewes were used together with the candidate females in one selection cycle. The values for survival rate, lambing interval, and litter size for the period during which breeding animals remained in the flock were taken from Welday et al. [10] and from monitoring data (record-keeping for 2 years) of the common Tigray highland sheep in Mekelle and Astbie-Wemberta representative districts. The conception rate of common Tigray highland sheep was derived from previous work on indigenous sheep breeds in Ethiopia [20].

**Table 1:** Biological, technical and economic parameters for four alternative breeding schemes for common Tigray highland sheep

Descriptors	Village-based breeding scheme		Central nucleus breeding scheme	
	Scheme 1	Scheme 2	Scheme 3	Scheme 4
Proportion of populations in the production unit	1.0	0.9905	0.95	0.95
Village nuclei		0.0095		0.02
Central nuclei			0.05	0.03
Number of villages in the production unit	7	127	127	127
Village nuclei	–	2		
Multiplier units				7
Lifetime use (years) of rams in the central nucleus			2	2
Lifetime use (years) of rams in villages	2	2	2	2
Lifetime use (years) of ewes in the central nucleus			7	7
Lifetime use (years) of ewes in villages	9.0	9.0		
Mating ration (F/M)—villages	35	35		
Mating ration (F/M)—central nucleus			25	25
Lambing interval (years)	0.66	0.66	0.66	0.66
Conception rate—central nucleus			0.90	0.90
Conception rate—villages	0.85	0.85		
Age at first lambing (years)				
Age of ewe at first lambing	1.5	1.5	1.5	1.5
Age of rams at first mating	0.70	0.70	0.70	0.70
Twinning rate	1.12	1.12	1.12	1.12
Survival of rams—villages	0.90	0.90		
Survival of rams—central nucleus			0.95	0.95
Survival of ewes—villages	0.90	0.90		
Survival of ewes—central nucleus			0.95	0.95
PWLS rate—village	0.89	0.89		
PWLS rate—central nucleus			0.95	0.95
Suitability for breeding	0.90	0.90	0.90	0.90
Fixed costs per ewe (Birr)	46.55	50.36	74.42	116.99
Variable costs per ewe (Birr)	25.37	28.25	40.42	74.76
Investment period (year)	10	10	10	10

*PWLS* pre-weaning lamb survival rate

The fixed costs include the salaries of the animal breeding experts for genetic evaluation, technical field assistants, and village coordinators, as well as the costs for maintaining nucleus flocks, data processing facilities, supplies and communications. Costs for animal identification, stationery, weighing scale and recording traits are included as variable costs. These costs vary between schemes depending on the nucleus sizes and selection activities across the different tiers of the schemes. The breeding program was planned for 10 years. The genetic parameters and economic values of the traits used to design the breeding schemes are in Table 2.

**Table 2:** Phenotypic standard deviations $\left( {{\upsigma }_{{\text{p}}} } \right)$, economic values, assumed heritabilities along the diagonal, genetic correlations (above the diagonal) and phenotypic correlations (below the diagonal) used in the simulated selection program for common Tigray highland sheep

Traits	Economic values (US $)	h^2^	SD $$\left( {{\upsigma }_{{\text{p}}} } \right)$$	SMW	ADG (kg) (3–6 months)	Litter size	PWLS
SMW	1.22	0.40	2.54	1.00	0.87	0.90	0.94
ADG (3–6 months)	1.07	0.26	0.02	0.43	1.00	0.89	0.23
Litter size	2.05	0.16	0.41	− 0.61	− 0.89	1.00	0.87
PWLS (0–3 month)	1.55	0.12	0.29	− 0.88	0.23	0.27	1.00

1USD = 29.54 Birr, in July 2019

*SMW* 6 month weight, *ADG* average daily gain (3–6 month), *PWLS* pre-weaning lamb survival (0–3 month), *SD* standard deviation, *h*^*2*^ heritability

### Genetic and phenotypic parameters

The phenotypic standard deviations, economic weight, estimates of the heritability of traits, genetic and phenotypic correlations used in the simulated selection program for common Tigray highland sheep are in Table 2. Phenotypic and genotypic variations (standard deviation) and their correlations with the breeding traits were obtained from on-farm monitoring of common Tigray highland sheep and the estimates of heritability were derived from the published works on indigenous sheep [21–23] since estimates of this genetic parameter are not available for common Tigray highland sheep breed. The economic weight for each trait was based on the breeding plan designed and computed by standardizing the values with the additive genetic standard deviation $\left( {{\upsigma }_{{\text{a}}} } \right)$ following the FAO guidelines [24]. From the monitoring study of common Tigray highland sheep, single-born lambs had a better survival rate compared to multiple-born lambs. Ewe prenatal nutrition competition is higher in the case of twins compared to single-born lambs and has a strong influence on lamb survival and growth [16]. Under feed shortage, ewes are hardly able to produce enough milk for their lambs which leads to poor preweaning growth performance of the lambs.

The marginal economic value for each trait was estimated as a change in profit resulting from an increase by one additive genetic standard deviation in the trait value due to selection by keeping the changes in other traits constant. Positive economic values indicated that genetic improvement in the traits would result in a positive effect on profitability. Additive genetic standard deviations $\left( {{\upsigma }_{{\text{a}}} } \right)$ of the traits were calculated as:

$$\upsigma _{{\text{a}}} = \surd {\text{h}}^{2} *\upsigma _{{\text{p}}} $$

where ${\text{h}}^{2}$ is the heritability and ${\upsigma }_{{\text{p}}}$ is the phenotypic standard deviation.

## Results

The predicted annual genetic gain for the breeding objectives was calculated by summing the genetic gains in the component traits (6-month weight, average daily body weight gain, litter size and pre-weaning lamb survival rate) weighted by their respective economic weights under four alternative breeding schemes (Table 3). The results show that the central nucleus schemes 3 and 4 resulted in faster genetic progress than the village-based breeding schemes 1 and 2. Scheme 3 scored the highest genetic gain (Table 3) followed by scheme 4, in which the open central nucleus breeding is linked with multiplier units. Among the four alternative breeding schemes, scheme 1 performed the least well in terms of annual genetic gain. This variation in annual genetic gain of 6-month weight, average daily gain (3–6 month), litter size and pre-weaning lamb survival rate among the four alternative breeding schemes depends on the selection method used and on the population size. Estimation of the genetic gain is known to be influenced by many factors, including population size, testing capacity, generation interval and possibly the evaluation techniques used [25, 26].

**Table 3:** Predicted genetic gains of the breeding traits under different breeding schemes

Parameters	Village-based breeding schemes		Central open nucleus-based breeding scheme	
	Scheme 1	Scheme 2	Scheme 3	Scheme 4
Breeding objective	10.657	13.732	30.403	29.063
SMW (kg)	0.2879	0.3708	0.8207	0.7849
ADG (3–6 month) (kg)	0.0010	0.0010	0.002	0.0019
Litter size	0.0030	0.0040	0.0086	0.0081
PWLS (0–3 months)	0.0020	0.0024	0.0054	0.0051

*SMW* 6 month weight, *ADG* average daily gain in kg (3–6 month), *PWLS* pre-weaning lamb survival rate (0–3 month)

The highest annual genetic gain was obtained for the 6-month weight trait, which improved by 0.821 kg and 0.785 kg annually under schemes 3 and 4, respectively. However, under the village-based breeding schemes 1 and 2, it improved only by 0.288 kg and 0.371 kg per year in the one-tier cooperative village-based breeding scheme and the two-tier cooperative village-based breeding scheme, respectively. The predicted higher annual genetic gain of 6-month weight trait in all breeding schemes is due to its higher phenotypic variation $\left( {{\upsigma }_{{\text{p}}} } \right)$, medium heritability and high phenotypic correlations with all the other breeding objective traits considered. Compared to the 6-month weight trait, annual genetic gains were lower for average daily body weight gain (3–6 months), litter size and pre-weaning lamb survival rate, which is due to their low phenotypic variation $\left( {{\upsigma }_{{\text{p}}} } \right)$ and to the low heritability of sheep litter size. The predicted lower annual genetic gain of average daily body weight gain (3–6 month) in common Tigray highland sheep (Table 3) is due to the low phenotypic variation $\left( {{\upsigma }_{{\text{p}}} } \right)$ of the common Tigray highland sheep population. Furthermore, most of the common Tigray highland sheep give birth during the wet season, when more feeds are available and lambs get more milk which leads to better growth performance while the growth of post-weaning animals depends on the smallholder farmers’ management practices. The availability of feed is limited during the dry season which affects the growth of the lambs compared to that during the wet season.

When ewes produce multiple lambs with better growth performance and with short lambing intervals, they are preferred by smallholder farmers and they achieve a faster genetic gain in the flock while their chances of survival decrease due to the competition between twins for consuming colostrum and milk from their dams. Higher pre-weaning lamb survival rate (the percentage of lambs born surviving up to weaning) is scored under the central open nucleus-based breeding scheme compared to the village-based breeding scheme (Table 3). Pre-weaning lamb survival rate depends largely on the mothering ability (nourishing potential) of the ewes whereas post-weaning lamb survival rate and growth performances depend largely on the small holder farmers’ traditional (no supplementation) management practices.

Table 4 presents the annual returns, costs and profit per ewe of the population for different alternative schemes, with the highest annual return found for the central nucleus-based breeding scheme 3 and the lowest returns for the village-based scheme 1 (Table 4). The annual costs were calculated per ewe per year in the whole population. Higher costs were scored in the village-based than in the central nucleus-based breeding scheme, which is attributed to the distribution of the costs of the central-based scheme throughout the whole population of about 11,005 breeding ewes. The village-based scheme with an improved lambing distribution (scheme-2) was more profitable than the village-based breeding scheme with the existing lambing distribution (scheme 1) but it was less profitable than schemes 3 and 4.

**Table 4:** Returns, costs and profits per ewe per year (US $) obtained from selection in common Tigray highland sheep using four alternative breeding schemes

Parameters	Village-based breeding scheme		Central open nucleus breeding scheme	
	Scheme 1	Scheme 2	Scheme 3	Scheme 4
Return/ewe/year (US $)	3.039	3.417	2.913	2.303
Cost/ewe/year (US $)	2.326	2.510	0.185	0.136
Fixed costs per ewe (US $)	1.480	1.577	0.118	0.074
Variable costs (US $)	0.846	0.933	0.067	0.062
Profit/ewe/year (US $)	0.713	0.908	2.728	2.167

1USD = 29.54 Birr, in July 2019

## Discussion

All breeding objective scenarios simulated as alternatives for small holder sheep producers had advantages in terms of genetic gain and profit. The results show that the genetic progress obtained in a village-based scheme with the existing lambing distribution over 1 year (scheme 1) was slow compared to the village-based scheme with an improved lambing distribution (scheme 2). The reason is that few selected candidates were available in each round of selection because of the dispersed lambing and of selection errors. Gizaw et al. [27] have already reported that low selection intensity, slow genetic progress and low impact on flock productivity and efficiency of genetic improvement are due to the small number of candidates for selection because of year-round lambing in the village-based sheep breeding program. However in another study, Gizaw et al. [3] predicted very close genetic gains from mass selection and best linear unbiased prediction (BLUP) estimated breeding values of selected candidates between village and community-based breeding schemes.

The three-tier breeding scheme (open breeding nucleus linked with multiplier units) produced a greater annual genetic gain and profit compared to schemes 1 and 2, but a slightly lower gain than the central open nucleus breeding scheme (scheme 3). The three-tier breeding scheme has an advantage over the central nucleus breeding which is due to a reduction in the time necessary for dissemination and adaptation to a particular production system and environment, which have considerable effects on the genetic improvement of the whole common Tigray highland sheep population in the region. Furthermore, linkage of a central nucleus breeding with smallholder farmers through multiplier units increases the accuracy of prediction of breeding values in the elite flock. According to Horton et al. [28], a benefit of 8 to 9% in dollars was achieved for meat and wool production by including multiplier flocks in the scheme compared to a two-tier sheep breeding scheme. However, the additional costs for establishing nuclei, infrastructure and human resources are difficulties to be faced in the open central nucleus breeding and multiplier units. Previous studies [27, 29] also reported that small herd sizes, the high cost of data recording and data processing, multiple and diverse breeding goals, poor infrastructure and institutional arrangements are the major technical limitations of conventional breeding schemes in smallholder production systems.

The choice of a breeding scheme should not be restricted to central nucleus- versus village-based schemes; a combination of both schemes needs to be considered, as well, to maximize genetic progress/profitability and facilitate the operation of breeding programs. Linkage of central nucleus breeding scheme with a smallholder farmer system through multiplier units (a total of seven village nuclei) serves as both multiplier and generators of genetic improvement for smallholder sheep producers. This scheme avoids the need to establish a large nucleus, which is costly and requires to limit geographically the central breeding nucleus. Introduction of biotechnology tools, particularly artificial insemination in the breeding program could facilitate the implementation of such breeding programs by overcoming the need for large nucleus flocks and the logistical limitations to transfer genetic gains across the breeding structure. However, this means solving the technical and institutional difficulties for developing efficient artificial insemination services, which have so far been a challenge in most developing countries [1].

The predicted genetic gains for 6-month weight, average daily body weight gain, litter size and pre-weaning lamb survival obtained in the current study are comparable with the results of Yohannes [30] and Hagos [31] for the Gumz sheep breed in the western lowland of Amhara and the Begait goat in the western zone of the Tigray region, respectively. Our results show that the genetic gain in average daily gain (3–6 months), litter size and lamb survival were low and similar between village-based schemes and central-based schemes. The low genetic gain in reproductive traits is attributed to their low heritability and phenotypic variation [23], and antagonism between litter size and survival rate. Similar findings were reported for litter size (0.0019–0.0021) and preweaning lamb survival rate (0.0015–0.0017) for the Menz, Afar, Bonga and Horro sheep breeds in Ethiopia [1, 32]. The comparison between the four alternative breeding schemes revealed that the discounted return for each breeding trait and the discounted profit per ewe per year were higher in the central open nucleus breeding scheme and three tier breeding program than in the cooperative village-based breeding schemes, which indicates that the benefit of these breeding schemes is greater (Fig. 1). However, some central nucleus-based breeding programs have failed under smallholder production systems [33, 34], which is due to the technical limitations and absence of infrastructures to support such breeding programs [35].

### Operational feasibility

Running central nucleus breeding schemes needs good infrastructures and technologies that have high establishment and operational costs, but in the case of whole common Tigray highland sheep populations in the Tigray region of northern, Ethiopia, it could result in increased genetic gain. The dissemination of the improved rams to smallholder farmers is difficult and less profitable because the overall production activities differ between the center and village levels. However, if smallholder farmers are strongly committed and supported by technologies such as artificial insemination and estrus synchronization, an improved genetic gain could be obtained within a short period of time. It is also recommended to distribute improved males to village cooperatives in a communal grazing system for communal uses because it will increase the mating ratio and the genetic and economic gains. Indeed, Gizaw et al. [36] noted that sharing genetically improved rams did increase the mating ratio, annual genetic progress and profitability of the Washera sheep breed. The operational feasibility of a village-based breeding scheme depends on the commitment of the villagers to participate in it. It can be more easily practiced in the case of small flock sizes, with a low initial cost and an overall traditional management compared to central nucleus breeding programs. However, the lack of accurate recording and current selection practices of the smallholder farmers affect the level of genetic gain that can be achieved. Kosgey and Okeyo [37] indicated that within-breed genetic improvement of small ruminants under smallholder production systems was constrained by both technical and infrastructural factors. Therefore, the modulation of an open central nucleus breeding system linked with smallholder farmers through multiplier units can bring rapid genetic gain of the whole common Tigray highland sheep population across different agro-ecological zones of the region.

## Conclusions

Both central nucleus-based and village-based breeding schemes were designed to improve the productivity of common Tigray highland sheep, and thus to increase its contribution to the livelihood of producers and the national economy. The two alternative breeding schemes evaluated in the current study were modeled on the basis of biological, economic and operational feasibilities. Our study revealed that open central nucleus-based breeding and linkage of a central nucleus with smallholder farmers through multiplier units resulted in higher genetic gain and economic efficiencies for all the breeding traits analysed compared to village-based breeding schemes. Based on the predicted genetic gain, we suggest that scheme 3 and scheme 4 are the most efficient schemes and that they need to be optimized for the genetic improvement and conservation of common Tigray highland sheep. Improved breeding management and technological interventions in village-based breeding schemes could result in more concentrated lambing periods and thus increase the selection intensity and genetic progress. Inter-linking of a central open nucleus breeding system with smallholder farmers through multiplier units in each district could rapidly address the flow of genetically superior animals to the whole common Tigray highland sheep population in their home tract. Further work on the application of a long-term breeding program such as cross breeding, genomic selection and updated estimates of genetic and economic values for the common Tigray highland sheep breed are also needed.

## Data Availability

The data that support the findings of this study are available from the corresponding author upon reasonable request.
